# Importance of Nutrition Visits After Gastric Bypass Surgery for American Veterans, San Francisco, 2004–2010

**DOI:** 10.5888/pcd11.140289

**Published:** 2014-12-24

**Authors:** Aung Zaw Win, Carol Ceresa, Anne L. Schafer, Peter Mak, Lygia Stewart

**Affiliations:** Author Affiliations: Carol Ceresa, Peter Mak, Clinical Nutrition, San Francisco Veterans Affairs Medical Center, San Francisco, California; Anne L. Schafer, Department of Medicine, University of California, San Francisco, Medical Service, San Francisco Veterans Administration Medical Center, San Francisco, California; Lygia Stewart, Department of Surgery, University of California, San Francisco, Surgical Service, San Francisco Veterans Administration Medical Center, San Francisco, California.

## Abstract

**Introduction:**

Nutrition counseling is important for veterans undergoing gastric bypass surgery. The aim of this study was to explore the relationship between the number of nutrition visits a patient attended and change in body mass index (BMI) after gastric bypass surgery for the veteran population.

**Methods:**

A retrospective study examined veterans (N = 79) who underwent Roux-en-Y gastric bypass surgery from June 2004 through July 2010. Spearman’s correlation and multivariate regression analysis were used to analyze data.

**Results:**

A significant correlation was found between the number of postoperative nutrition visits and the change in postsurgery BMI at 2 years (Spearman’s ρ = 0.21; *P* = .017). After adjusting for age, sex, and race, the association between postsurgery nutrition visits and BMI change persisted (β = 0.255; 95% confidence interval, 0.015–0.581; *P* = .039).

**Conclusion:**

Veterans with more nutrition visits following Roux-en-Y gastric bypass surgery experienced greater declines in BMI. This finding underscores the importance of the dietitian on the bariatric surgery team.

## MEDSCAPE CME

Medscape, LLC is pleased to provide online continuing medical education (CME) for this journal article, allowing clinicians the opportunity to earn CME credit.

This activity has been planned and implemented in accordance with the Essential Areas and policies of the Accreditation Council for Continuing Medical Education through the joint sponsorship of Medscape, LLC and Preventing Chronic Disease. Medscape, LLC is accredited by the ACCME to provide continuing medical education for physicians.

Medscape, LLC designates this Journal-based CME activity for a maximum of 1 **AMA PRA Category 1 Credit(s)™**. Physicians should claim only the credit commensurate with the extent of their participation in the activity.

All other clinicians completing this activity will be issued a certificate of participation. To participate in this journal CME activity: (1) review the learning objectives and author disclosures; (2) study the education content; (3) take the post-test with a 75% minimum passing score and complete the evaluation at www.medscape.org/journal/pcd (4) view/print certificate.

**Release date: December 24, 2014; Expiration date: December 24, 2015**

### Learning Objectives

Upon completion of this activity, participants will be able to:

- Describe the association between the number of nutrition visits a patient attended and change in body mass index after gastric bypass surgery, based on a retrospective study of veterans
- Discuss findings of other recent studies regarding the association between the number of nutrition visits a patient attended and change in body mass index after gastric bypass surgery
- Distinguish the role of nutrition counseling in patients undergoing gastric bypass surgery

**EDITORS**

Rosemarie Perrin, Editor, *Preventing Chronic Disease*. Disclosure: Rosemarie Perrin has disclosed no relevant financial relationships.

**CME AUTHOR**

Laurie Barclay, MD, Freelance writer and reviewer, Medscape, LLC. Disclosure: Laurie Barclay, MD, has disclosed no relevant financial relationships.

**AUTHORS AND CREDENTIALS**

Disclosures: Aung Zaw Win, MD, MA, MPH; Carol Ceresa, MHSL, RD; Anne L. Schafer, MD; Peter Mak, BS, RD; Lygia Stewart, MD have disclosed no relevant financial relationships.

Affiliations: Aung Zaw Win, Carol Ceresa, Peter Mak, Clinical Nutrition, San Francisco Veterans Affairs Medical Center, San Francisco, California; Anne L. Schafer, Department of Medicine, University of California, San Francisco, Medical Service, San Francisco Veterans Administration Medical Center, San Francisco, California; Lygia Stewart, Department of Surgery, University of California, San Francisco, Surgical Service, San Francisco Veterans Administration Medical Center, San Francisco, California.

## Introduction

From 2009 through 2010, the prevalence of obesity in the United States was 35.5% among adult men and 35.8% among adult women (1). Over the next 2 decades, obesity is expected to increase an additional 33% and severe obesity by 130% (2). The United States spends over $190 billion annually on health care directly related to obesity (3). Among veterans receiving care through the Veterans Health Administration (VHA), 73% of men and 68% of women are overweight or obese (4). The incidence of obesity has increased substantially among US veterans (5). Obesity is associated with diseases in all of the organ systems, and gastric bypass surgery can prevent secondary complications of obesity.

Surgical treatment of obesity is more effective and produces longer lasting outcomes than medication therapy or counseling (6). More than 250,000 bariatric procedures were performed in the United States in 2008 (7), and that number will continue to increase. Roux-en-Y gastric bypass (RYGB) surgery has become the gold standard among bariatric surgeries, and it is the most commonly performed bariatric procedure in the United States and all over the world (7,8). Gallagher et al reported that the cost of RYGB surgery at the VHA is offset by reduction of health care costs within the first year after surgery (9). The procedure is also reported to increase life expectancy by an average of 7.1 years (10). Moreover, weight loss surgery has been reported to ameliorate chronic medical conditions such as diabetes and improve quality of life (11,12). In veterans, long-term follow-up has confirmed significant and durable weight loss with marked improvement in comorbidities (5). Independent studies have reported significantly greater weight loss after RYGB than with vertical banded gastroplasty (13). The maximum weight loss after RYGB occurs at 2 years after the surgery, and after 2 years, up to 25% of lost weight can be regained (14,15). Studies have followed the RYGB outcomes up to 15 years after the surgery (16). However, few long-term follow up studies on results of RYGB have been conducted for the VHA patient population (14).

The role of the registered dietitian nutritionist is to perform dietary assessments, to evaluate for nutritional deficiencies, and to provide counseling to help patients meet postsurgery weight loss goals (17). Dietitians are part of the multidisciplinary bariatric team. Expert guidance and visits to dietitians are part of the surgical process. Veterans are an underprivileged and disenfranchised population, and they suffer from greater socioeconomic and health disparities than the general population (4). The aim of this study was to explore the relationship between the number of nutrition visits a patient made and change in body mass index (BMI) after gastric bypass surgery for the veteran population. 

## Methods

This project was reviewed and approved by the San Francisco Veterans Administration Medical Center Institutional Review Board and was performed in accordance with the ethical standards laid down in the 1964 Declaration of Helsinki and its later amendments or comparable ethical standards. Before analysis, patient records and information were de-identified to ensure anonymity. Data from patients who underwent RYGB at our institution from June 2004 through July 2010 were collected from the computerized patient record system. A total of 79 patients who had RYGB during the study period constituted the study population. Date of surgery, age, sex, race, height, BMI, number of nutrition encounters during the 2 years after surgery, and weight before and 2 years after surgery were recorded. Patients’ weights (in kg) were measured by health care professionals during clinic visits. BMI was calculated as weight (in kg) divided by height (in m^2^). Change in BMI was calculated by subtracting presurgery BMI from BMI 2 years postsurgery.

SPSS version 20.0 (SPSS, Inc) was used to analyze the data. Spearman’s correlation was used to determine the association between number of nutrition visits and BMI change. Multivariate linear regression was used to determine the effects of nutrition visits, sex, race, and age on BMI change. A *P* value of <.05 was considered significant for this study. 

## Results

Characteristics of patients are shown in the Box. The mean number of nutrition encounters during the 2 years postsurgery was 6.44 visits (standard deviation [SD], 5.36; range, 1–29). The average BMI change was a decrease of 14.66 (SD, 6.26; range, 1.3–38.1) at 2 years postsurgery. There was a significant relationship between the number of postsurgery nutrition encounters and the change in BMI 2 years after surgery (Spearman’s ρ = 0.21; *P* = .017). After adjusting for age, sex, and race, there was an association between postsurgery nutrition visits and BMI change (β = 0.255; 95% CI, 0.015–0.581; *P* = .039) (Table).

**Box. Characteristics of the Study Population (N = 79), Effect of Post-Surgery Nutrition Follow-up Visits on Body Mass Index Change Among Veterans, San Francisco, 2004–2010**

Age, y

Mean, 51.35; standard deviation, 8.73; range, 27–67

Sex

Male, 65 (82%)

Female, 14 (18%)

Race/ethnicity

White, 55 (70%)

Black, 15 (19%)

Hispanic, 9 (11%)

**Table:** Multivariate Linear Regression Analysis of Factors Affecting PostSurgery Weight Loss Among Veterans (N = 79) for the Effect of Nutrition Visits on Postsurgery Body Mass Index Change, San Francisco, 2004–2010

Variable	β	*P* value	95% Confidence Interval
Postsurgery nutrition visits	0.255	. 04	0.015 to 0.581
Age	−0.091	.45	−0.238 to 0.107
Sex	−0.004	.96	−3.181 to 3.342
Race	0.006	.97	−1.74 to 1.683

## Discussion

In this study, patients attending more nutrition visits following surgery experienced greater declines in BMI. These nutrition visit-specific results are consistent with those of Compher et al, who examined the relationship between clinical visits and post–gastric bypass weight loss and found that the odds of weight loss increased 2.8-fold with each clinical visit at 2 years following surgery (18). Lier et al found that regular attendance to postoperative nutrition counseling visits was associated with weight loss 1 year after gastric bypass surgery (19). Furthermore, Shen et al reported that patient follow-up played a significant role in the amount of weight lost after bariatric surgery (20). Patients with no follow-up visits were 4.6 times more likely to regain weight (21).

Nutrition care before surgery, during the hospital stay, and after surgery is important for the quality of life of the patient and the success of the surgery. There are no set evidence-based guidelines on the postsurgery nutrition follow-up schedule, which differs from institution to institution (22). Current VA guidelines recommend that patients have 2 to 4 nutrition visits presurgery and 2 to 3 visits during hospital stay. Furthermore, nutrition appointments should be scheduled at 1 to 2 weeks, 4 to 6 weeks, 3 months, 6 months, and 12 months after surgery, and then annually afterwards. Patients require a lifetime of nutritional follow-up to have a good quality of life (20), because bariatric surgery patients are at risk of malnutrition and micronutrient deficiencies following surgery.

Nutrition visits are helpful for bariatric surgery patients. Dietary counseling improved nutrient intake of gastric bypass surgery patients (23). Freire et al stated that lack of nutritional counseling follow-up was significantly associated with regaining weight (24). Through frequent contact dietitians can detect challenging situations such as increased food urges, inactive lifestyle, and eating fast foods, all of which can contribute to regaining weight (17). Regular nutrition visits can also aid detection and prevention of micronutrient deficiencies and severe malnutrition. Nutritional deficiencies are very common after RYGB despite supplementation with standard doses of multivitamins. The common micronutrient deficiencies that occur after surgery are potassium, B1, B3, B6, B12, folate, iron, copper, zinc, selenium, and vitamins A, D, E, K, and C (22). Thus, nutrition visits can prevent regaining weight, osteomalacia, protein–calorie malnutrition, and, most of all, micronutrient deficiencies (22). Dietitians can also help prevent dumping syndrome in patients by giving appropriate dietary advice. In addition, contact with medical professionals is a source of motivation for patients (18).

This study had limitations. It was a retrospective study, and VHA patients are mostly white, male, and over the age of 50. Moreover, the sample size was small. In an older patient population, weight loss can be generally slower because of a lower metabolic rate from reduced muscle mass. Admiraal et al found that whites had more weight loss than African Americans after bariatric surgery (25). Whites made up the majority in this study population. Most published studies on bariatric surgery outcomes consist of 70% to 80% women (26). Bekheit et al found no difference in weight loss between men and women after RYGB (27). Still et al mentioned in their study that age and sex do not correlate with postoperative weight loss (28). Odom et al found no relationship between weight loss and race (21). As in the results obtained by Bekheit et al, Still et al, and Odom et al, our study found no correlation between postsurgery BMI change and age, race, or sex.

Freire et al stated that postsurgery nutritional follow-up visits can decrease dramatically as time passes (24). A significant number of patients will not comply with regular follow-up unless they are prompted to do so (29). Continued support through communication and monitoring is needed to sustain weight loss. Patient follow-up can be a challenge for veterans. Bariatric surgery is offered in only 12 medical centers in the VHA system, and some patients have to travel long distances to these centers for surgery. After discharge, they return to their primary care locations for follow-up. Thus, some veteran patients can be lost to follow-up, which appears to be a major barrier to postsurgery weight loss (18). Veterans, in general, are more obese or overweight, and they suffer from more illnesses than the general population (4). There is also a higher prevalence of substance abuse and mental health problems among veterans (30). Adams et al reported that veterans with a history of substance abuse had low weight loss 2 years after surgery (10). Most VHA patients are over the age of 50, have attained low education levels, and have low socioeconomic status (5). Moreover, lower socioeconomic status of veterans can limit access to exercise facilities and food choices that may have an effect on total weight loss (14).

Registered dietitians and nutritionists have an integral role in helping patients meet their weight loss goals following bariatric surgery. According to the VA guidelines, RYGB patients should have had at least 6 nutrition visits at 2 years after surgery. Strategies need to be developed to optimize the number of nutrition visits and to encourage veterans to come to all scheduled nutrition appointments. In this study, veterans attending more postsurgery nutrition visits experienced greater weight loss in the 2 years after surgery. Prospective studies may confirm the association between BMI change and the number of nutrition visits.

## Figures and Tables

**Table:** 

**1. The activity supported the learning objectives.**				
**Strongly Disagree**				**Strongly Agree**
1	2	3	4	5
**2. The material was organized clearly for learning to occur.**				
**Strongly Disagree**				**Strongly Agree**
1	2	3	4	5
**3. The content learned from this activity will impact my practice.**				
**Strongly Disagree**				**Strongly Agree**
1	2	3	4	5
**4. The activity was presented objectively and free of commercial bias.**				
**Strongly Disagree**				**Strongly Agree**
1	2	3	4	5

## Post-Test Information

To obtain credit, you should first read the journal article. After reading the article, you should be able to answer the following, related, multiple-choice questions. To complete the questions (with a minimum 75% passing score) and earn continuing medical education (CME) credit, please go to http://www.medscape.org/journal/pcd. Credit cannot be obtained for tests completed on paper, although you may use the worksheet below to keep a record of your answers. You must be a registered user on Medscape.org. If you are not registered on Medscape.org, please click on the "Register" link on the right hand side of the website to register. Only one answer is correct for each question. Once you successfully answer all post-test questions you will be able to view and/or print your certificate. For questions regarding the content of this activity, contact the accredited provider, CME@medscape.net. For technical assistance, contact CME@webmd.net. American Medical Association's Physician's Recognition Award (AMA PRA) credits are accepted in the US as evidence of participation in CME activities. For further information on this award, please refer to http://www.ama-assn.org/ama/pub/about-ama/awards/ama-physicians-recognition-award.page. The AMA has determined that physicians not licensed in the US who participate in this CME activity are eligible for **AMA PRA Category 1 Credits**™. Through agreements that the AMA has made with agencies in some countries, AMA PRA credit may be acceptable as evidence of participation in CME activities. If you are not licensed in the US, please complete the questions online, print the AMA PRA CME credit certificate and present it to your national medical association for review.

## Post-Test Questions

### Article Title: Importance of Nutrition Visits After Gastric Bypass Surgery for American Veterans, San Francisco, 2004–2010

**CME Questions**

1. Your patient is a 62-year-old man for whom Roux-en-Y gastric bypass surgery is being considered. According to the retrospective study by Win and colleagues, which of the following statements about the association between the number of nutrition visits a patient attended and change in body mass index (BMI) after gastric bypass surgery is ***correct***?
  - The association between the number of postsurgery nutrition visits and the change in BMI at 2 years after surgery was not statistically significant
  - Adjustment for age, gender, and race abolished the association between postsurgery nutrition visits and change in BMI
  - The findings support the importance of the dietitian on the bariatric surgery team
  - During the 2 years after surgery, the mean number of nutrition encounters was 3 visits
2. According to other recent studies, which of the following statements about the association between the number of nutrition visits that a patient attended and change in BMI after gastric bypass surgery is ***correct***?
  - Other studies negate the findings of the present study
  - Compher and colleagues found that the odds of weight loss increased by 50% with each clinical visit at 2 years after surgery
  - Lier and colleagues found no association between regular attendance to postoperative nutrition counseling visits and weight loss 1 year after gastric bypass surgery
  - A study showed that patients with no follow-up visits after gastric bypass surgery were 4.6 times more likely to regain weight
3. According to the retrospective study by Win and colleagues, and other recent research, which of the following statements about the role of nutrition counseling in patients undergoing gastric bypass surgery is ***correct***?
  - Current Veterans Affairs guidelines recommend nutrition visits at 1 to 2 weeks, 4 to 6 weeks, 3 months, 6 months, and 12 months after surgery, and annually thereafter
  - Bariatric surgery patients are not at increased risk for malnutrition after surgery
  - Bariatric surgery patients are not at increased risk for micronutrient deficiencies after surgery
  - Dietitians play no role in preventing dumping syndrome

**Evaluation**
